# Pregnancy and delivery after mid-urethral sling operation

**DOI:** 10.1007/s00192-020-04497-w

**Published:** 2020-08-25

**Authors:** Sari A. Tulokas, Päivi Rahkola-Soisalo, Mika Gissler, Tomi S. Mikkola, Maarit J. Mentula

**Affiliations:** 1 Department of Obstetrics and Gynaecology, University of Helsinki and Helsinki University Hospital, PO Box 140, FI-00029 Helsinki, HUS Finland; 2 Finnish Institute for Health and Welfare (THL), 00300 Helsinki, Finland; 3 Department of Neurobiology, Care Sciences and Society, Karolinska Institute, SE-171 77 Stockholm, Sweden; 4 Folkhälsan Research Center, Biomedicum, Haartmaninkatu 8, 00290 Helsinki, Finland

**Keywords:** Mesh tape, Mid-urethral sling, Pregnancy, Stress urinary incontinence, TOT, TVT, TVT-O

## Abstract

**Introduction and hypotheses:**

There is no consensus regarding pregnancy after mid-urethral sling (MUS) operation, and some clinicians recommend postponing the MUS operation if a woman considers further pregnancies or routinely suggest cesarean section as the delivery method after MUS operations. Our primary aim was to assess the risk for stress urinary incontinence (SUI) re-procedure after delivery in women with a MUS operation prior to pregnancy. We also analyzed SUI re-visits and MUS-related complications during pregnancy and postpartum.

**Methods:**

We conducted a register-based case-control study of women with a MUS operation in Finland during 1996–2016. We identified 94 cases with a subsequent pregnancy and 330 controls without subsequent pregnancies matched by age, operation type and year.

**Results:**

The median follow-up time was 10.7 years (IQR 7.1–13.7). The number of SUI re-procedures did not differ between the cases (*n* = 3, 3.2%) and controls (*n* = 17, 5.2%; OR 0.6, 95% CI 0.2–2.1). There was no significant difference in re-visits for stress or mixed urinary incontinence between the cases (*n* = 23, 24.5%) and controls (*n* = 86, 26.1%; OR 0.9, 95% CI 0.5–1.6), but 35% of the re-visits in the case group occurred already before the delivery after MUS. The rate of vaginal delivery was lower after MUS operation (57%) than in deliveries before MUS (91%, *P* < 0.001).

**Conclusions:**

Pregnancy after MUS did not increase the odds for SUI re-procedure or re-visit. Considering on our results, future pregnancy does not need to be viewed as an absolute contraindication for MUS operation.

**Electronic supplementary material:**

The online version of this article (10.1007/s00192-020-04497-w) contains supplementary material, which is available to authorized users.

## Introduction

Stress urinary incontinence (SUI) is a common disorder in women. Approximately 10–14% of women will have an operation for SUI during their lifetime [1, 2], and the mid-urethral sling (MUS) is considered the gold standard surgical treatment for SUI. Women are commonly advised to postpone surgical treatment for SUI until childbearing has been completed [3] because of the fear of SUI relapse or complications during pregnancy or delivery [3]. It has also been commonly recommended that cesarean section should be the mode of delivery after a MUS operation [4]. Thus, SUI operations are relatively uncommon among women younger than 40, even though the prevalence of severe SUI is around 10% among women between 25 and 49 years of age [1, 5].

There is no guideline or clinical consensus concerning the MUS operation and future pregnancies. Only in two cohort studies and smaller case series has pregnancy after MUS operation been assessed showing that pregnancy after MUS did not increase the risk for SUI relapse or SUI re-operation [10, 11].

In our register-based case-control cohort study, we assessed the incidence of urinary incontinence and re-operation for SUI in women with a previous MUS operation. We also report the number of potentially MUS-related complications during pregnancy and postpartum subsequent to a MUS operation.

## Materials and methods

We conducted a case-control study of women who had a retropubic (RP-MUS) or transobturator mid-urethral sling (TO-MUS, including inside-out TVT-O and outside-in TOT) operation in Finland between 1996 and 2016. As cases, we identified all women who had had a pregnancy ending in delivery after the index operation, and for each case, we selected up to four controls without subsequent deliveries. Controls were matched with cases by age (± 2 years), MUS type (retropubic vs. transobturator) and MUS operation year (± 2 years). The follow-up continued until the end of 2017.

**Fig. 1:**
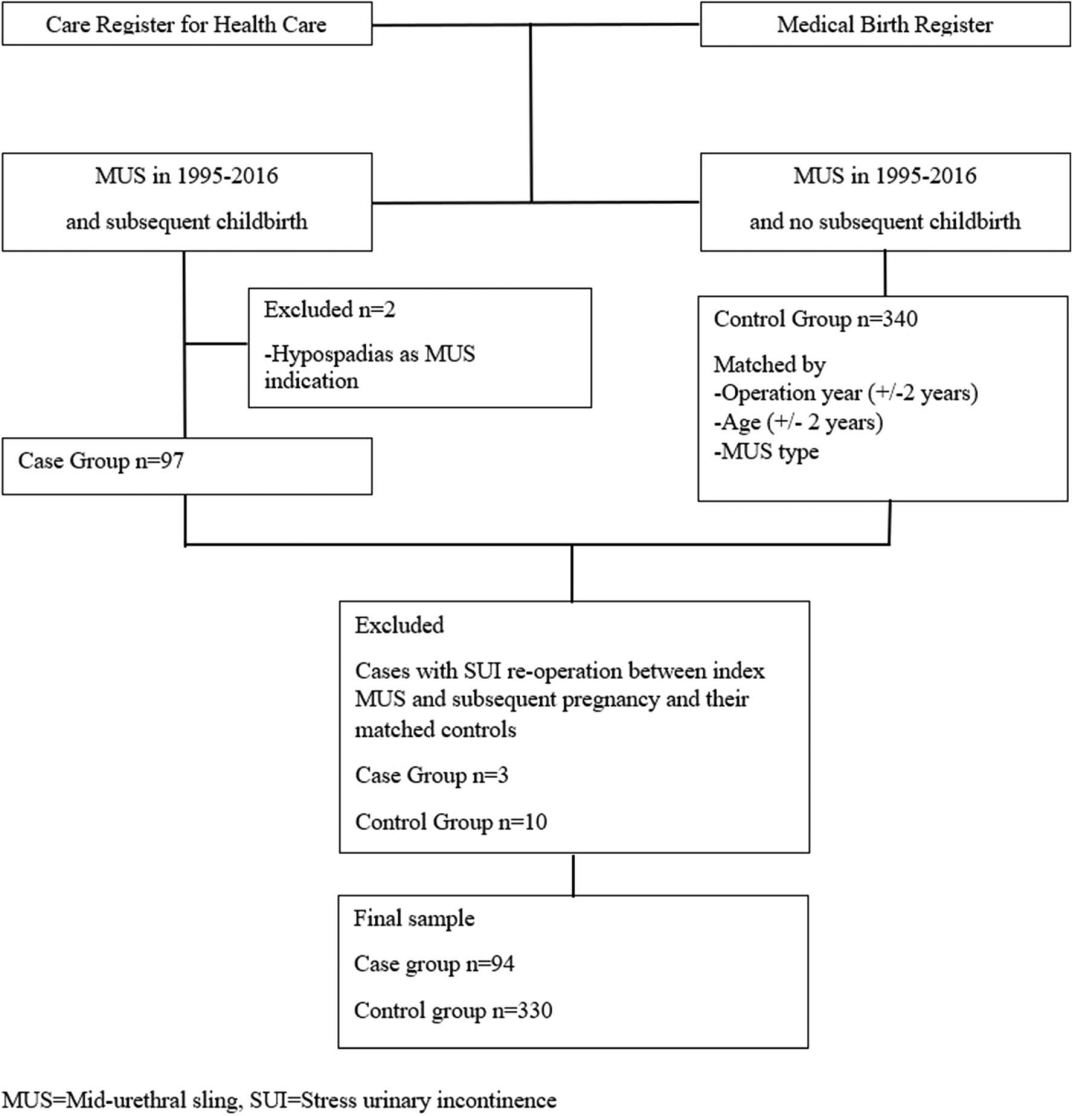
Flowchart of sampling process

The cases and their controls were identified by linking two national registers: the Care Register for Health Care (Care Register), in which all health care providing institutes in Finland are required by law to report all their specialized medicine patient visits, and the Medical Birth Register, in which all deliveries in Finland are recorded including information on the delivery, the delivered babies and their mothers (Fig. 1). We identified the sample by searching all visits with the Nordic Medico-Statistical Committee Classification of Surgical Procedures (NCSP) operation codes for MUS operation (LEG10 for retropubic MUS, LEG12 for outside-in transobturator MUS and LEG13 for inside-out transobturator MUS). Then, women with a delivery after the MUS operation were identified by a search of the Medical Birth Register. From the remaining women without a subsequent delivery, up to four matched controls per each case were randomly selected. The preoperative incontinence type for the sample women was considered as SUI if the ICD-10 diagnosis code for the operation was N93.3, mixed urinary incontinence (MUI) if it was N39.4 and unknown if any other diagnosis code was used.

As cases, we identified 99 women who had a MUS procedure from 1995 to 2016 and a subsequent pregnancy ending in delivery (Fig. 1). Two of them were excluded from further analysis because they had the MUS procedure for hypospadias, and they were younger than 18 during the time of the procedure. Another three were excluded from further analysis because they had a SUI re-operation between the index MUS and the subsequent pregnancy. Thus, the case group consisted of 94 women. We identified 330 matched controls who had a MUS procedure from 1995 to 2016 but no subsequent pregnancies. Due to the matching criteria, fewer than four controls were available for some cases: the number of identified matching controls was 4 for 78 cases, 3 for 2 cases, 2 for 4 cases, 1 for 4 cases and 0 for 6 cases. Due to the selection criteria of age and MUS type, we did not have enough controls in the database to select from.

New incontinence visits and re-procedures were identified from the Care Register and Register of Primary Health Care visits (Register of PHC) by using appropriate ICD-10 codes for urinary incontinence and NCSP codes for SUI procedures (ICD-10 codes for SUI re-visit: stress urinary incontinence N39.3 and mixed urinary incontinence N39.4, NCSP codes for SUI re-procedure; see Supporting Information Table S1). All public health care-providing institutes in Finland are required by law to report admission and discharge dates, and all diagnosis and procedure codes of all in- and out-patient visits. Visits in specialized medicine are reported to the Care Register, and visits in general medicine are reported to the Register of PHC. Therefore, our data include all incontinence visits and re-procedures performed in the public sector in Finland. Of all MUS procedures in Finland, 98.4% were performed in the public sector during our study period (Finnish Institute for Health and Welfare). Visits within the first 60 days after the index operation were considered control visits and were not included in the analysis.

Pregnancy and post-partum complications were identified from the Care Register, the Register of PHC and the Medical Birth register. We identified all visits from 315 days before to 42 days after the postoperative delivery from the Care Register and the Register of PHC and considered them as complications if the visit included the diagnostic code for urinary incontinence, urinary tract infection, erosion, abdominal or pelvic or perineal pain, acute pain, urinary retention, dysuria, pollakiuria, other difficulties to urinate and perineal lacerations during vaginal delivery (see Supporting Information Table S2 for the detailed list of ICD-10 diagnostic codes and NCSP-codes used). We also identified visits for SUI and MUI for women who had not had previous visits for incontinence between the index operation and the postoperative pregnancy.

The main outcome was new SUI procedures, which were defined as a visit recorded in the Care Register with a NCSP code for MUS, urethral bulking injection, colposuspension or bladder neck needle suspension (see the detailed NCSP codes used in Supporting Information Table S1). Secondary outcomes were re-visits for SUI or MUI after the index operation, the effect of the delivery mode on the risk for a new SUI procedure and complications during pregnancies and post-partum after MUS operation defined as diagnostic codes reported in the Medical Birth Register as illnesses during pregnancy.

The treatment of SUI in Finland follows the National Guideline [6]. The first-line treatment options for SUI are conservative, including pelvic floor muscle training, but operative treatment is indicated if conservative treatment fails. The first-line operative treatment option has been MUS, especially RP-MUS, since the late 1990s. Other operations, such as Burch colposuspension, have been seldom used [1]. National MUS practices are uniform because of a structured training program when RP-MUS was first introduced [7]. By the end of 1999 in Finland, MUS operations were performed in 40 hospitals [7], and by 2018 they were performed in 25 public hospitals (Finnish Institute for Health and Welfare).

BM SPSS Statistics 25 was used for statistical analysis. To compare groups, we used the Student’s t-test for continuous variables and chi-squared or Fisher’s exact test, when appropriate, for categorical variables. To calculate confidence intervals, we used Clopper-Pearson for binomial variables and Student’s t-test for continuous variables. We used the odds ratio and Cox regression to analyze risk factors for a new SUI procedure.

The Finnish Institute for Health and Welfare of Finland authorized the use of the data from the Care Register, the Medical Birth Register and the Register for PHC (THL/945/5.05.00/2017). The register authorities assessed the ethics of the study, and as no contact with the subjects was included, the study was exempted from evaluation by an Ethics Committee.

## Results

In our study population RP-MUS was more common than TO-MUS (Table 1). The only demographic difference between the groups was parity; the case women were more often both primiparas and multiparas (3 or more deliveries) than the control women (*P* < 0.001). The median follow-up time was 10.7 years (IQR 7.1–13.7).

**Table 1:** Sample demographics

	Cases (94)	Controls (330)	*P*
Incontinence type, *n* (%)			0.5
SUI	81 (86)	267 (81)	
MUI	5 (5)	23 (7)	
Unknown or other	8 (9)	40 (12)	
Index operation, *n* (%)			1.0
RP-MUS	61 (65)	213 (65)	
TO-MUS	33 (35)	117 (35)	
Age at index operation, median (IQR)	35 (32–38)	36 (33–38)	0.08
Parity before index operation, *n* (%)			< 0.001
0	9 (10)	13 (4)	
1–2	57 (61)	279 (85)	
3 or more	28 (30)	38 (12)	
Index operation year, *n* (%)			0.08
1997–1999	2 (2)	0 (0)	
2000–2004	29 (31)	98 (30)	
2005–2009	36 (38)	133 (40)	
2010–2014	26 (28)	89 (27)	
2015–2016	1 (1)	10 (3)	
BMI at start of last pregnancy before index MUS			0.9
Median (IQR)	23 (21–25)	23 (21–26)	
Unknown, *n* (%)	63 (70)	217 (66)	
Follow-up time, median in years (IQR)	10.8 (7.2–13.9)	10.6 (7.1–13.7)	
Mode of deliveries before index operation, *n* (%)			0.1
Only vaginal	77 (91)	280 (88)	
One or more cesarean sections	8 (9)	31 (10)	
Unknown	0 (0)	6 (2)	
Number of deliveries after index MUS			
1	85 (90)	–	
2	9 (10)	–	
Mode of delivery after index operation, *n* (%)			
Vaginal	54 (57)	–	
Elective cesarean section	24 (26)	–	
Urgent or emergency cesarean section	16 (17)	–	
Difference compared with delivery mode before index operation			< 0.001
Time to delivery after index MUS, median in years (IQR)	2.6 (1.6–4.6)	–	0.7

SUI = stress urinary incontinence, MUI = mixed urinary incontinence, RP-MUS = retropubic mid-urethral sling,

TO-MUS = transobturator mid-urethral sling, BMI = body mass index, MUS = mid-urethral sling

New SUI procedures were performed in 3 cases (3.2%) and on 17 controls (5.2%, Table 2). There was no difference in the number of new procedures for SUI between the cases and controls (*P =* 0.6) or the median time between the index MUS and the re-procedure (4.4 and 4.6 years, respectively, *P* = 1.0). None of the tested variables (subsequent pregnancy, incontinence type, operation type and parity) affected the risk for SUI re-procedure (Table 3). Of the three cases who had a SUI re-procedure, two had a vaginal delivery and one had an elective cesarean section.

**Table 2:** Re-procedures and re-visits for stress urinary incontinence after mid-urethral operation

	Cases (94)	Controls (330)	OR (95% CI)	*P*
SUI re-procedure, *n* (%)	3 (3.2)	17 (5.2)	0.6 (0.2–2.1)	0.6
RP-MUS	1 (33.3)	9 (52.9)		
TO-MUS	0	4 (23.5)		
Bulking injection	2 (66.7)	3 (17.6)		
Other vaginal operation	0	1 (5.9)		
Re-procedures between index MUS and subsequent delivery included	6 (6.2)	17 (5.0)	1.2 (0.5–3.3)	0.6
Time to re-operation, median in years (IQR)	4.4 (2.7-NA)	4.6 (2.3–7.6)		0.5
SUI or MUI re-visit, *n* (%)	23 (24.5)	86 (26.1)	0.9 (0.5–1.6)	1.0
Re-visits between index MUS and subsequent delivery	15 (65.2)	–		
Re-visit incontinence type				
SUI	14 (60.9)	57 (66.3)		
MUI	9 (39.1)	29 (33.7)		
Time to re-visit, median in years (IQR)	2.6 (0.0–6.0)	0.7 (0.0–3.0)		0.04

SUI = stress urinary incontinence, MUI = mixed urinary incontinence, RP-MUS = retropubic mid-urethral sling, TO-MUS = transobturator mid-urethral sling, MUS = mid-urethral sling

**Table 3:** Risk factor analysis for stress urinary incontinence re-procedure after mid-urethral sling operation

Univariate analysis	HR	95% CI, *P* = 0.05		*P*
Postoperative delivery				0.4
No deliveries	1 (ref)			
Postoperative delivery	0.6	0.2	2.1	
Incontinence type				0.2
SUI	1 (ref)			
MUI or other	1.9	0.7	4.9	
Operation type				1.0
RP-MUS	1 (ref)			
TO-MUS	1.0	0.4	2.6	
Parity before index operation				0.2
Nulliparous	1 (ref)			
1–2 deliveries	0.5	0.1	2.1	
3 or more deliveries	0.1	0.01	1.6	
Multivariate analysis	HR	95% CI, *P* = 0.05		*P*
Postoperative delivery adj. with incontinence type and parity				0.3
MUI or other (ref: SUI)	2.0	0.7	5.3	
1–2 deliveries (ref: nulliparous)	0.4	0.1	2.3	
3 or more deliveries (ref: nulliparous)	0.1	0.01	1.6	
Postoperative delivery (ref: no delivery)	1.5	0.4	5.4	

SUI = stress urinary incontinence, MUI = mixed urinary incontinence, RP-MUS = retropubic mid-urethral sling

TO-MUS = transobturator mid-urethral sling, HR = hazard ratio

Re-visits for SUI and MUI were equally common in the case group (*n* = 23, 25%) and control group (*n* = 86, 26%; OR 0.9, 95% CI 0.5–1.6) (Table 2). Among the cases, 63% of the re-visits (*n* = 15) occurred already before pregnancy. In both the case and control groups, the majority (61% and 66%) of the re-visits were due to SUI.

Of the 94 first deliveries after MUS, 54 (57%) were vaginal deliveries, 24 (26%) were elective cesarean sections, and 16 (17%) were urgent or crash cesarean sections. The number of elective cesarean sections was significantly higher in women with MUS operation prior to pregnancy compared with their last delivery before the MUS operation (*P* < 0.001). The proportion of cesarean sections did not vary during the follow-up period. Nine case women had two deliveries after MUS; none of them had SUI re-operations.

During the pregnancy or postpartum after MUS, 18 cases (18.6%) had a visit for urinary tract infection, urinary incontinence, or pelvic or perineal or lower abdominal pain (Table 4). There were no visits for urinary retention or other urinary symptoms or erosion.

**Table 4:** Complications during pregnancy and post-partum after mid-urethral sling operation

Complication, *n* (%)	Pregnancy	Postpartum
Incontinence	4 (4)	0 (0)
Stress urinary incontinence	3 (3)	0 (0)
Mixed urinary incontinence	1 (1)	0 (0)
Urinary tract infection	2 (2)	2 (2)
Pain	8 (9)	4 (4)
Pelvis or perineum	1 (1)	0 (0)
Lower abdomen	3 (3)	2 (2)
Other abdominal pain	3 (3)	1 (1)
Acute pain	0 (0)	1 (1)
Urinary retention, dysuria and other urinary symptoms	0 (0)	0 (0)
Sling-specific complications	0 (0)	0 (0)
Perineal laceration during delivery		3 (3)
Grade I		2 (2)
Grade II		1 (1)
Grade III and IV		0 (0)

## Discussion

We show that in women who had a pregnancy ending in delivery after a MUS operation, the rate of SUI re-procedures did not differ from that of controls without a subsequent pregnancy (3.2% vs. 5.2%, respectively, *P =* 0.6). This result is in line with previous studies [10, 11]. The long follow-up time, median 10.7 years, assured that we were able to find the majority of the re-procedures, since they mostly took place within the first postoperative years [8, 9]. The median time from MUS to childbirth was 2.6 years, and thus the follow-up was still long enough to show most of the relapses.

In previous studies assessing the effect of childbirth after MUS, the rate (18% and 22%) [10, 11] of women with symptomatic SUI are in line with the number of re-visits in our study. This, at least in part, may reflect the rate of various SUI symptoms. In a previous study, the SUI re-procedure rate was assessed as a secondary outcome, and this number (5.6%) is also comparable with our findings [11]. The original MUS operations were developed and studied in a Nordic co-work [12], and thus it is likely that similar technique and indications in the Nordic countries explain the comparable results. However, smaller case series from other countries also show MUS to be effective after subsequent childbirth [13–15].

We show that delivery after MUS was not a significant risk factor for SUI relapse, being in line with previous studies [10, 11]. However, the end point in our study was a SUI re-procedure or re-visit instead of subjective cure after the follow-up measured by questionnaires. In contrast, we were able to evaluate the exact timing of SUI relapses and identify those that occurred before the subsequent pregnancy.

The analysis of secondary end points, complications of MUS during pregnancy, delivery or post-partum period showed no urinary retention or dysuria. The number of urinary tract infections during pregnancy and post-partum was also low (4%), and no recurrent urinary tract infections were found. In previous studies, a relatively low 1.4% de novo voiding dysfunction needing intermittent catheterization was detected, and 2.8% reported recurrent urinary tract infections [11]. These numbers are reassuring, showing that MUS does not cause high risk for urinary tract symptoms during pregnancy and post-partum. However, 12 women (13%) in our case group had visits during pregnancy or post-partum because of pain in the abdomen or perineum. We were unable to assess whether these visits for pain were directly MUS related and whether the pain was later resolved. Other studies have not reported chronic pelvic pain [11], but further studies are needed since pain is one of the major concerns associated with MUS use.

There is no consensus on the mode of delivery in women with a previous MUS operation, and many doctors have recommended cesarean section in fear of recurrent SUI after vaginal delivery [4]. Even though we could not assess the reasons for choosing the delivery mode, it is likely that MUS has been an indication for some cesarean sections since elective cesarean sections were significantly more frequent after the MUS operation, even though 82% of parous case women had only vaginal deliveries before the index MUS. The total section rate after MUS in our data was 42%, which is lower (58% and 54%) than in previous studies [10, 11]. Although up to 77% of women in a previous study [10] reported that MUS was an indication for cesarean section, according to our and previous data [10, 11], vaginal delivery does not affect MUS results, and thus the mode of delivery should be decided by obstetric indications.

The main strength of our study is the comprehensive national compulsory health care registries. These registries provide a relatively large population-based sample without a selection bias. Another strength is the ability to identify exact time points of the SUI re-procedures and re-visits and assess only those that occurred after the delivery subsequent to the index MUS. We were also able to identify all the health care visits during the pregnancy after MUS and post-partum. The long follow-up time can be considered a strength as well.

As a limitation in our study, we acknowledge that we were unable to detect recurrent SUI and other complications unless the women decided to seek help from a doctor. Using SUI re-procedures as the main end point also produced only few end point events, which resulted in a wide confidence interval and small power. However, we consider re-visits and particularly re-procedures as robust end points, because they are not subject to recall bias, for example, and they reflect clinically relevant rates of SUI recurrence well. Unfortunately, we were unable to match the cases and controls according to their preoperative parity. However, parity was not a significant risk factor for a SUI re-procedure in our data, and other studies also show conflicting results [10, 11]. We were also unable to report the BMI at the time of the index MUS; however, there was no significant difference in BMI between the cases and controls at the beginning of the last pregnancy before the index operation. We also acknowledge that we were unable to determine the indication for postoperative cesarean delivery; however, we were able to identify the previous delivery method as a reference point and identify elective cesarean sections, which could be more likely chosen because of a previous MUS operation than urgent and crash cesarean sections. We were also unable to assess if the complications during pregnancy and post-partum were MUS-related, but the overall number of complication visits was low. Even though SUI re-procedure is a reliable main end point, it produced only few events; thus, our study was under-powered to analyze other risk factors than subsequent pregnancy.

To conclude, our data show that in women with a previous MUS operation, pregnancy and delivery do not increase the odds for SUI re-procedure or an incontinence-related re-visit. Furthermore, previous MUS operation does not increase the rate of MUS-related complications during pregnancy, delivery or post-partum. Thus, our results suggest that future pregnancy plans do not need to be considered as an absolute contraindication for MUS operation.

## Electronic supplementary material

ESM 1 (DOCX 16 kb)

ESM 2 (DOCX 16 kb)
