# Supplementary material for: Pregnancy and delivery after mid-urethral sling operation
Source: Int Urogynecol J. 2020 Aug 25;32(1):179–86. doi: 10.1007/s00192-020-04497-w (PMC7788014; doi:10.1007/s00192-020-04497-w)
Supplement: Supplementary file 1 — (DOCX 16 kb) [file 192_2020_4497_MOESM1_ESM.docx]

**Appendix 1: Operation codes according to the Nordic classification of surgical procedures used to identify visits for stress urinary incontinence re-procedure**

| **Code** | **Description** |
| --- | --- |
| KDG* | Operations on urethra and bladder neck for urinary incontinence |
| KDG00 | Retropubic suspension of urethra (Eponym: Marshall-Marchetti-Krantz) |
| KDG01 | Percutaneous endoscopic retropubic suspension of urethra |
| KDG10 | Abdominovaginal suspension of bladder neck (Eponym: Stamey,  synonyms: Endoscopic suspension of bladder neck, Needle urethropexy) |
| KDG20 | Abdominal colposuspension (Eponym: Burch) |
| KDG21 | Percutaneous endoscopic colposuspension  (Includes: Preperitoneal colposuspension using laparoscope) |
| KDG30 | Suprapubic sling urethrocystopexy |
| KDG31 | Percutaneous endoscopic suprapubic sling urethrocystopexy |
| KDG40 | Suprapubic urethrocystopexy |
| KDG41 | Percutaneous endoscopic suprapubic urethrocystopexy |
| KDG43 | Transobturatorial sling urethrocystopexy |
| KDG50 | Transabdominal plastic repair of pelvic floor for urinary incontinence |
| KDG60 | Implantation of adjustable expander around bladder neck |
| KDG96 | Other operation on urethra or bladder neck for incontinence |
| KDG97 | Other percutaneous endoscopic operation on urethra or bladder neck for incontinence |
| KDV20 | Submucous urethral injection |
| KDV22 | Transluminal endoscopic submucous urethral injection |
| LEG00 | Vaginal urethrocystorrhaphy (Eponyms: Kelly, Kennedy etc.) |
| LEG10 | Vaginal urethrocystopexy (Eponym: TVT) |
| LEG12 | Vaginal transobturatorial urethropexy, outside-in (Eponym: TOT) |
| LEG13 | Vaginal transobturatorial urethropexy, inside-out (Eponym: TVT-O) |
| LEG96 | Other vaginal operation for incontinence |
