# Supplementary material for: Pregnancy and delivery after mid-urethral sling operation
Source: Int Urogynecol J. 2020 Aug 25;32(1):179–86. doi: 10.1007/s00192-020-04497-w (PMC7788014; doi:10.1007/s00192-020-04497-w)
Supplement: Supplementary file 2 — (DOCX 16 kb) [file 192_2020_4497_MOESM2_ESM.docx]

**Appendix 2: diagnostic codes (ICD-10) used to identify complications during pregnancy and post-partum**

| **Code** | **Description** |  |
| --- | --- | --- |
| **Urinary tract infections** | |  |
| N10 | Acute pyelonephritis |  |
| N30 | Cystitis |  |
| N30.0 | Acute cystitis |  |
| N30.1 | Interstitial cystitis (chronic) |  |
| N30.2 | Other chronic cystitis |  |
| N39.0 | Urinary tract infection, site not specified |  |
| O23.0 | Infections of kidney in pregnancy, unspecified trimester |  |
| O23.1 | Infections of bladder in pregnancy |  |
| O23.2 | Infections of urethra in pregnancy |  |
| O23.3 | Infections of other parts of urinary tract in pregnancy |  |
| O23.4 | Unspecified infection of urinary tract in pregnancy |  |
| O86.2 | Urinary tract infection following delivery |  |
| **Urinary incontinence** | |  |
| N39.3 | Stress incontinence (female) (male) |  |
| N39.4 | Other specified urinary incontinence |  |
| **Dysuria** |  |  |
| R30 | Pain associated with micturition |  |
| R30.0 | Dysuria |  |
| R30.1 | Vesical tenesmus |  |
| R30.9 | Painful micturition, unspecified |  |
| **Retention of urine** | |  |
| R33 | Retention of urine |  |
| R33.8 | Other retention of urine |  |
| R33.9 | Retention of urine, unspecified |  |
| **Other urinary symptoms** | |  |
| R35 | Polyuria |  |
| R35.0 | Frequency of micturition |  |
| R35.1 | Nocturia |  |
| R35.8 | Other polyuria |  |
| R39.1 | Other difficulties with micturition |  |
| **Pain** |  |  |
| R10.2 | Pelvic and perineal pain |  |
| R10.3 | Pain localized to other parts of lower abdomen |  |
| R10.4 | Other and unspecified abdominal pain |  |
| R52.0 | Acute pain |  |
| R52.1 | Chronic intractable pain |  |
| **Perineal laceration during delivery** | |  |
| O70 | Perineal laceration during delivery |  |
| O70.0 | First degree perineal laceration during delivery |  |
| O70.1 | Second degree perineal laceration during delivery |  |
| O70.2 | Third degree perineal laceration during delivery |  |
| O70.3 | Fourth degree perineal laceration during delivery |  |
| O70.4 | Anal sphincter tear complicating delivery, not associated with third degree laceration |  |
| O70.9 | Perineal laceration during delivery, unspecified |  |
